# Patient satisfaction with services in physiotherapy clinics: a cross sectional study at teaching hospitals in Klang Valley

**DOI:** 10.1186/1471-2458-12-S2-A15

**Published:** 2012-11-27

**Authors:** Mohamad Nasaruddin Mahdzir, Aniza Ismail

**Affiliations:** 1 Centre for Graduate Studies, Faculty of Medicine, Universiti Teknologi MARA, 47000 Sg. Buloh, Malaysia; 2 Department of Community Health, Universiti Kebangsaan Malaysia Medical Centre, Jalan Yaacob Latiff, 56000 Kuala Lumpur, Malaysia

## Background

The physiotherapy service is a new and emerging field in most hospitals throughout Malaysia as well as internationally. As such, there is still a lack of scientific research and reporting about the finding of service quality studies in Physiotherapy Clinics at teaching hospital settings in Malaysia. It is timely and relevant then that research is conducted on the quality of services in teaching hospitals in terms of physiotherapeutic system which includes examination, treatment, advice and instructions for the purpose of connection with movement dysfunction, bodily malfunction, physical disorder, disability, healing and pain from trauma and disease. This study was to determine the level of patient satisfaction and its contributing factors as well as to determine the quality of services in Physiotherapy Clinics at Teaching Hospitals in Klang Valley.

## Materials and methods

The cross sectional study was conducted from March until July 2011 among outpatients who attended Physiotherapy Clinic at two teaching hospitals (311 patients) in Klang Valley. Patients were recruited using simple random sampling technique and data were collected by using the self-administered Service Quality (SERVQUAL) Questionnaire.

## Results

About 62.4% patients at Physiotherapy Clinics in Teaching Hospitals had their expectations met. The ‘Assurance’ in Teaching Hospitals showed the highest satisfaction score in SERVQUAL components. The ‘Caring Services’ in `Corporate Culture’ component showed the lowest satisfaction score of all dimensions. The socio-demographic factors which significantly influenced patient satisfaction include age, education status, occupational status and number of visit. There were significant differences in patient satisfaction in the following SERVQUAL components (Tangibles (P=0.00), Reliability (P=0.01), Responsiveness (P=0.00), Assurance (P=0.03), Empathy (P=0.03)), Outcome component (Treatment (P=0.00) and Corporate Culture components (Caring Services (P=0.02), Team Work (P=0.00), Professionalism (p=0.00). The predictors for overall patients’ satisfaction in Teaching Hospitals were not influenced by factors studied (P>0.05).

## Conclusions

Levels of patient’s satisfaction were found to be moderate for teaching hospitals. The dimension related to ‘Caring Services’ should be given focus in order to improve the level of patients’ satisfaction at Physiotherapy Clinics.

